# A Practical Treatise on Mechanical Dentistry

**Published:** 1880-12

**Authors:** 


*A Practical Treatise on Mechanical Dentistry.*—By Joseph Richardson, D. D. S., M. D. Third Edition, revised and enlarged, with one hundred and eighty-five illustrations. Publishers: Lindsay and Blackiston, Philadelphia, 1881.

In the preparation of this new edition of a standard work, new and improved methods and appliances of recent introduction and important supplementary matter in connection with the older ones have received careful attention. Additional practical details relating to Continuous Gum Work are embodied in the new volume, also chapters relating to Celluloid and Gold Alloy Cast Base, after Reese's method. A distinct chapter has also been assigned to Porcelain Teeth in connection with Carved Block Work; also a separate chapter to the method of attaching teeth to a metalic plate base by means of celluloid or rubber. Considerable additions have also been made to the chapter on Pivoting Artificial Crowns, and other matter excluded which judgement and experience have proven to be impracticable, and useless, among the latter being the cast aluminum plate base, which the author regards as being practically useless.

The illustrations are finely executed and the style of the work is creditable to its well known publishers.

